# Between Anxiety and Adaptation: Children’s and Parents’ Experiences with Botulinum Toxin Treatment in Cerebral Palsy

**DOI:** 10.3390/jcm14093164

**Published:** 2025-05-02

**Authors:** Rannei Sæther, Siri Merete Brændvik, Ann-Kristin Gunnes Elvrum

**Affiliations:** 1 Regional Centre for Child and Youth Mental Health and Child Welfare, Department of Mental Health, Faculty of Medicine and Health Sciences, Norwegian University of Science and Technology, 7130 Trondheim, Norway; 2 Department of Rehabilitation Science and Health Technology, Faculty of Health Sciences, OsloMet—Oslo Metropolitan University, P.O. Box 4, St. Olavsplass, 0130 Oslo, Norway; 3 Department of Neuromedicine and Movement Science, Faculty of Medicine and Health Sciences, Norwegian University of Science and Technology, 7130 Trondheim, Norway; siri.merete.brandvik@ntnu.no (S.M.B.); ann-kristin.elvrum@ntnu.no (A.-K.G.E.); 4 Clinic of Rehabilitation, St. Olav’s Hospital, Trondheim University Hospital, 7130 Trondheim, Norway

**Keywords:** cerebral palsy, BoNT-A treatment, procedural experiences, child-centered care

## Abstract

**Background/Objectives**: This study explores how children with cerebral palsy (CP) and their parents experience botulinum toxin type A (BoNT-A) treatment, focusing on emotional and procedural challenges and communication within the triad of children, parents, and healthcare providers. **Methods**: This qualitative sub-study was conducted within the WE-study, a randomized controlled trial on BoNT-A effects in children with CP. Semi-structured interviews with 20 parents and 18 children (aged 4–15 years, GMFCS I–II) were thematically analyzed. **Results**: Three themes were identified: Preparing for the treatment, Being in the moment, and Adapting after treatment. Pre-procedural anxiety was common, with children describing nervousness or physical discomfort in the days before the treatment. During the procedure, pain management and sedation choices influenced children’s experiences, with healthcare providers being the primary source of information. After treatment, some children experienced temporary walking instability, but most quickly resumed daily activities. Communication primarily occurred between healthcare providers and each party individually, rather than through a triadic interaction. **Conclusions**: BoNT-A treatment involves both emotional distress and adaptation. Strengthening child-inclusive communication, structured preparation, and collaboration within the triad may improve treatment experiences and better align care with child-centered principles. Future research should explore strategies to enhance child involvement in repeated treatments.

## 1. Introduction

Cerebral palsy (CP) is the leading cause of physical disability in children, characterized by impairments in movement and posture resulting from early brain injury [1]. While approximately 70% of children with CP can walk independently [2], many experience challenges such as impaired balance [3], increased energy expenditure [4], and fatigue [5], which may further restrict their participation in physical and social activities [6]. Spasticity, which is present in more than 80% of children with CP, can lead to limited range of motion and reduced motor function [7]. Accordingly, spasticity management is an essential part of rehabilitation management.

A commonly used intervention for managing spasticity is botulinum toxin type A (BoNT-A) injections. In Norway, more than 50% of children with CP receive BoNT-A treatment [8], with the calf muscles being the most frequently targeted to improve gait patterns among independent walkers [7]. While BoNT-A has been shown to reduce spasticity and improve equinus gait, its overall effects on walking and participation remain uncertain [9]. Recent studies have also raised concerns about potential long-term impacts on muscle morphology [10]. In addition, pain and anxiety related to the injection procedures have been highlighted as significant challenges, particularly in pediatric patients [11,12].

Much of the existing research on BoNT-A treatment has focused on quantitative assessments of its physiological effects, while fewer studies have explored caregivers’ and children’s lived experiences [13]. Previous qualitative research suggests that caregivers frequently observe beneficial effects, including less spasticity, enhanced motor abilities, and signs of increased activity and happiness in their children [14,15]. However, they also describe the treatment as painful and distressing, raising concerns about the balance between benefits and drawbacks [16].

There has been a growing acceptance of the importance of including children’s perspectives in research, reflecting a shift from a healthcare provider-dominated approach to a more family- and patient-centered model [17]. In this context, the principles of child-centered care (CCC) emphasize the importance of acknowledging children’s voices and experiences in healthcare decisions [18,19]. However, previous studies on BoNT-A treatment have primarily focused on caregivers’ perspectives, with limited attention to children’s own experiences [14,15,16].

Therefore, the aim of this study is to explore both children’s and parents’ perspectives on BoNT-A treatment, providing insights into their experiences of the procedure. As far as we are aware, this is the first study to explore both perspectives, providing insights that may inform clinical practice and promote child-centered care.

## 2. Materials and Methods

### 2.1. Design

This study is a qualitative sub-study within the Walking Easier with CP (WE) study, a multicenter double-blind, placebo-controlled randomized controlled trial (RCT; gov registration: NCT02546999) investigating the effects of BoNT-A injections in the calf muscles in children with CP [20,21]. While the main RCT focused on objective clinical outcomes, this sub-study explored children’s and parents’ experiences of BoNT-A treatment through qualitative interviews.

### 2.2. Participants and Recruitment

Participants were recruited from the Norwegian cohort of the WE study [21] and included children and adolescents aged 4.0 to 17.5 years with spastic unilateral (UL) or bilateral (BL) CP, classified at Gross Motor Function Classification System (GMFCS) level I or II. This qualitative sub-study is based on the same sample as our previous mixed-methods study, which explored the correspondence between expected, perceived, and measured effects of BoNT-A treatment [13].

A purposeful sampling strategy was used to ensure diversity in age, gender, and treatment experiences. Both children and one of their parents were invited to participate; however, children younger than six years were not interviewed due to anticipated limitations in their ability to provide meaningful reflections. The final sample was considered to provide sufficient information power [22] in relation to the study aim, and thematic analysis was conducted following the principles of reflexive thematic analysis [23].

### 2.3. Clinical Setting

This multicenter study was conducted in a clinical setting, involving three of Norway’s four health regions. BoNT-A injections were administered using ultrasound guidance, enabling precise visualization of the target muscle and accurate needle placement. Participants received treatment under local anesthesia, with the option of conscious sedation using oral or nasal benzodiazepines if requested by the participants or their caregivers. Sedation procedures followed standard guidelines at each participating center. For details on dosage and other procedural aspects, refer to the study protocol [20].

### 2.4. Ethical Considerations

The WE study received approval by the Regional Ethical Committee for Medical Research in North Norway (2013/1195). Written informed consent was obtained from all parents, and children provided verbal or written consent based on their age and cognitive abilities.

### 2.5. Data Collection

Semi-structured face-to-face interviews were conducted in a comfortable setting by the first author (RS), an experienced physiotherapist with 30 years of clinical work with children with CP, as well as experience in conducting interviews. The interviews were carried out at two time points: baseline (before treatment) and four weeks post-treatment (P1), on the same day as the clinical hospital-based assessments, except for two follow-up interviews conducted via telephone at P1. All interviews were audio-recorded and transcribed verbatim for analysis.

To encourage open and meaningful responses, children and parents (either the mother or father) were interviewed separately using open-ended questions. The interviews began with a general question: “Can you tell me about the activities you/your child enjoy?” The interview guide then covered key themes, including participants’ knowledge about BoNT-A treatment, such as what they knew about the procedure, the information they had received beforehand, their understanding of how BoNT-A works, and how the treatment was conducted, including preparation and execution. Participants were also asked to describe how they or their child experienced the treatment itself, followed by reflections on how they or their child felt after the procedure (see Table 1). For the child interviews, the questions were adapted to suit the individual child’s age, developmental level, and communication abilities. Throughout the interviews, participants were encouraged to discuss any additional relevant topics that emerged naturally in the conversation.

### 2.6. Data Analysis

The transcribed interviews were analyzed using reflecive thematic analysis (TA), following the six-step framework developed by Braun and Clarke [23]. This approach allows for a systematic yet flexible exploration of participants’ experiences, capturing both shared and individual perspectives while ensuring a structured interpretation of the data [23].

The analysis began with familiarization with the data, where the first author (RS) and a medical doctor (IT) carefully read and re-read the transcribed interviews to gain an in-depth understanding of the material. Initial coding was then conducted inductively, with meaningful text segments systematically identified and labeled across the dataset. These codes were generated without a pre-existing coding framework.

In the next step, codes were grouped into potential themes by identifying patterns and connections between different aspects of the participants’ experiences. These themes were then reviewed and refined to ensure they accurately represented the data and maintained coherence across the dataset. This process involved discussions between RS and IT, where discrepancies in coding were resolved, and subthemes were identified to provide a more nuanced understanding.

Once the themes were finalized, the next step involved defining and naming themes, ensuring that each theme was clearly described and reflected the core experiences captured in the interviews. Finally, the results were synthesized into a coherent narrative, integrating supporting quotes from participants to illustrate key findings and enhance credibility.

### 2.7. Interpretative Framework

The findings were interpreted according to the child-centered care (CCC) framework [18], which emphasizes agency, participation, impact, decision-making, and communication in children’s healthcare experiences. This framework provided a lens to evaluate how the BoNT-A treatment process aligned with child-centered principles, particularly regarding information provision, decision-making involvement, and emotional support.

To ensure conceptual clarity, Table 2 presents an overview of the five CCC principles, as described by Carter et al. [18]. These principles highlight key aspects of child-centered care and serve as a foundation for the discussion.

## 3. Results

### 3.1. Study Participants

A total of 20 children and adolescents who participated in the WE study, along with one of their parents, were recruited for the qualitative interviews. The sample consisted of 10 girls and 10 boys aged 4 to 15 years and included 12 mothers and 8 fathers. Two children under 6 years were not interviewed. Most of the children had unilateral (UL) CP, GMFCS level I (see Table 3). Twelve (n = 13) of the children (65%) had received BoNT-A treatment earlier.

### 3.2. Themes

In the data analysis, the following three themes were identified: 1. Preparing for the treatment. 2. Being in the moment. 3. Adapting after treatment (Table 4).

*Preparing for the treatment* captures the anticipation leading up to the procedure, where children navigate a balance between fear and familiarity while also struggling with varying levels of understanding and access to information. *Being in the moment* explores how children experience pain management and the injection itself, including their perception of control and sensory responses. Finally, *Adapting after treatment* highlights the bodily and functional adjustments children undergo, along with how rewards and rituals support their coping process.

Together, these themes provide insight into children’s experience of BoNT-A treatment, revealing both the challenges and adaptive strategies that shape how children and parents engage with the procedure.

In our presentation of the themes, we first highlight the voices of the children and parents within each category, followed by a brief interpretation that outlines the theme’s contribution to knowledge.

### 3.3. Prepearing for the Treatment

#### 3.3.1. Between Fear and Familiarity

The anticipation of BoNT-A treatment was marked by anxiety and apprehension among children. Many described the days leading up to the procedure as stressful, with one child stating, “I have been nervous every time” (C02). For some, the anxiety manifested bodily, as one child recalled: “The last day before the treatment, I was shaky and didn’t want to eat” (C19). Parents also observed this distress, noting behavioral changes such as disrupted sleep patterns, “She was a little anxious and didn’t sleep well” (M19).

While most children reported being apprehensive about the treatment, a few described feelings of acceptance or resignation. One child said, “It’s okay. I have done it so many times that I have gotten used to it” (C18). A parent similarly noted, “He doesn’t look forward to it, but it’s fine” (F18), suggesting that repeated exposure to the procedure may reduce anticipatory anxiety over time. Still, for others, the emotional burden persisted, as another parent observed, “He can get a bit sad and have tears in his eyes at night” (M02).

These findings highlight how repeated medical procedures can contribute to both heightened stress responses and, in some cases, adaptive coping mechanisms over time.

#### 3.3.2. Navigating Information

The way children and parents processed information about the treatment varied. Some children found medical explanations difficult to understand. One child admitted, “She explained a little, but I don’t really understand. I don’t get the nurse language” (C19). This statement highlights the challenge of translating complex medical terminology into age-appropriate language. Others demonstrated a clearer understanding of the procedure’s intended effects, “Botox makes my muscle more relaxed, so it is easier to use, but it takes time to work” (C05).

Parents also had varying experiences with the information they received. While some felt well-informed, others had lingering concerns, “We have read a bit ourselves. Botox is a nerve toxin that temporarily relaxes the muscle” (M01). One parent, however, was surprised upon learning more about the nature of BoNT-A: “I later heard that Botox isn’t that great. I have questions about what it really is and its side effects” (M05).

These experiences suggest that tailored communication is essential to ensure both children and parents feel adequately informed and prepared for the procedure.

### 3.4. Being in the Moment

#### 3.4.1. Managing Pain

Pain management strategies varied, and children had different preferences regarding sedation. Some preferred to remain as alert as possible, while others opted for medication to ease the process. One child explained, “I didn’t want full anesthesia, so I just drank something to make me relaxed” (C05). Another struggled with the pre-medication, “I couldn’t drink the liquid medicine, it tasted too bad, so I had to take tablets instead” (C10).

Parents emphasized the importance of giving children some degree of control over their sedation options. One parent noted, “She was happy that she opted out of the sedation that made her feel indifferent because she didn’t want to be groggy afterwords” (M13). Another parent similarly stated, “She chose to have nitrous oxide instead of other sedation. It was important for her to have control” (M05), adding that providing explanations and reassurance further alleviate distress, “She felt safe because they explained everything well” (M05). Others noted challenges with sedation, “He hated the medicine and refused to drink it, which made things harder” (M12).

These accounts suggest that allowing children some autonomy in their pain management choices foster a greater sense of control and security during medical interventions.

#### 3.4.2. Experiencing the Injection

The injection process itself was described as a challenging experience by most of the children. Some reported that they felt only minor sensations at first, but discomfort increased with subsequent injections, “The first three injections didn’t hurt, but the fourth one did. It was a weird, ticklish feeling” (C05). Others found the injections more distressing, “It was painful. Annoying. It felt like it was bursting inside” (C13).

For some children, sedation reduced or eliminated memory of the event: “I don’t remember it, but they say I got it. I think I got some memory-forgetting medicine” (C10). Parents, however, still observed signs of discomfort. One recalled, “It was quite fast. They just injected it all at once. Even though she doesn’t remember, it must have hurt” (M06).

The variation in children’s and parents’ experiences of pain and discomfort suggests that sedation and pain management strategies should be individualized to optimize comfort and minimize distress.

### 3.5. Adapting After Treatment

#### 3.5.1. Regaining Stability

Following the treatment, children commonly reported temporary instability when walking. One child described their experience, saying, “It felt a bit strange to walk. I was a little off balance” (C17). Another noted, “Right after, I could trip and fall because the medicine felt weird” (C18). Parents observed similar effects, with one commenting, “She was walking, but she was very unsteady. It looked like she had been drinking” (M03).

Despite this initial walking instability, likely caused by both the injection and the premedication, children adapted quickly. “He limped a bit but still managed to ride his bike as soon as he got home” (F18), one parent shared, indicating that the instability was temporary.

These findings suggest that while BoNT-A treatment may cause short-term disruptions in motor function, most children demonstrated resilience and adaptability in returning to daily activities.

#### 3.5.2. Coping Through Rewards

Despite the challenges, children often found ways to cope by focusing on post-treatment rewards. “Straight to the toy store!” (C06), one child exclaimed, emphasizing the role of incentives in making the experience more tolerable. Another reflected, “I get to have my mom all to myself that day” (C19), suggesting that the emotional support and attention from parents played a key role in their coping strategies.

This highlights the importance of both tangible and emotional reinforcement in helping children process and manage medical procedures.

To sum up, the results illustrate the complex interplay among children, parents, and healthcare providers in shaping the BoNT-A treatment experience. The pre-treatment phase was often marked by anxiety, shaped by varying access to tailored information and families’ perceived ability to influence the process. During treatment, pain management and sedation played a key role, with some children expressing a desire for greater control. Post-treatment, children adapted to temporary walking instability with resilience, though experiences varied.

**Figure 1:**
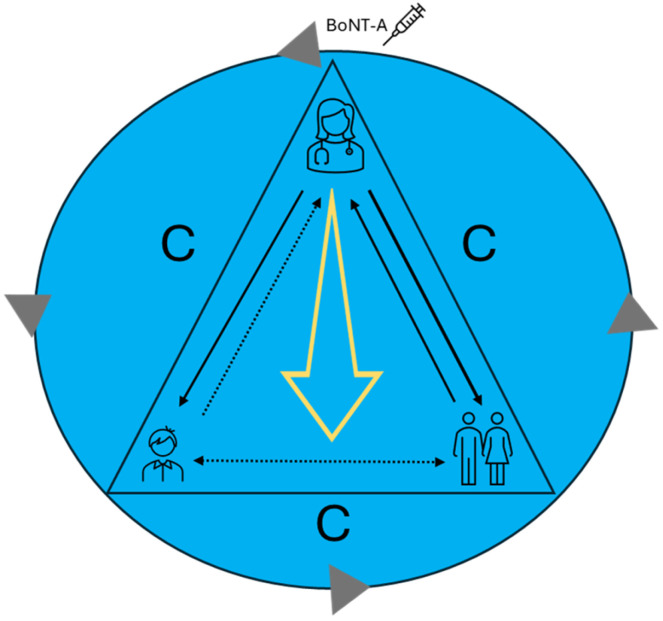
Communication within the triad of children, parents, and healthcare providers.

These findings suggest that while parents provided emotional and practical support, healthcare professionals remained the primary source of information. Although communication likely took place with all parties present, it appears that children were often not actively involved or did not fully understand the information provided. As a result, communication within the triad often occurred in a way that was directed more toward either parents or children individually, rather than through joint discussions (Figure 1). This may have limited the opportunity to establish a shared understanding of the treatment, potentially making it more challenging for parent–child collaboration in preparing for and managing the treatment. These findings highlight a need to explore ways to better support mutual understanding and coping strategies.

## 4. Discussion

The results from this study reveal how anticipation, bodily sensations, and meaning-making in interactions with healthcare providers influenced children’s experiences with BoNT-A treatment. These factors appeared to shape how children managed anxiety before the procedure, their responses to pain management, and their adaptation post-treatment. Furthermore, the findings may suggest that interaction primarily occurred between healthcare providers and each party (child or parent) individually, rather than through a triadic interaction. In the following, these findings will be discussed in relation to CCC and previous research.

### 4.1. Discussion in Light of CCC

Applying the CCC framework, as delineated by Carter et al. [18], provides valuable insight into how BoNT-A treatment can be better aligned with children’s needs and experiences. The five CCC principles—*agency*, *participation*, *impact*, *decision*-*making*, *and communication*—offer a structured lens for interpreting the findings of this study.

*Agency* was reflected in how children approached sedation choices, drawing on past experiences to shape their preferences. Although major decisions were made by parents and healthcare providers, these moments allowed children some control over their treatment, aligning with CCC’s emphasis on recognizing children as active participants.

However, agency extends beyond specific choices and is fundamentally linked to children’s rights and participation in healthcare. Recognizing children as agentic beings means moving beyond occasional involvement in decision-making to foster an environment where they are seen as capable social actors. Structured opportunities for children to understand, question, and contribute to decisions about their treatment could strengthen their role as active stakeholders in their healthcare experience.

*Participation* was evident in how children engaged with the treatment process, where some expressed a desire to be included and asked for information, while most remained passive. Healthcare providers were the primary source of information, while parents played a more active role in seeking details. This indicates that while participation was possible, it was largely dependent on external facilitation rather than children’s own initiative.

According to CCC, participation should go beyond passive involvement to ensure that children’s perspectives and preferences are actively elicited. CCC highlights that participation is shaped not only by children’s willingness but also by external factors such as communication strategies and parental mediation. Creating structured opportunities for children to express concerns, ask questions, and discuss procedural preferences could make participation more meaningful and integrated into care.

*Impact* was evident in how children’s anxiety before treatment affected their well-being, often leading to sleep disturbances and loss of appetite. These findings highlight the emotional burden of the procedure and align with CCC’s recognition that healthcare experiences can have both immediate and lasting effects on children’s well-being. While distress was acknowledged, it was not always systematically addressed.

On the other hand, impact should extend beyond recognizing distress to actively shaping practice. CCC emphasizes that children’s experiences should inform and improve healthcare routines both at an individual and systemic level. Strengthening structured preparation routines and implementing feedback mechanisms where children’s experiences directly influence procedural adjustments could help reduce uncertainty and stress.

*Decision-making* was primarily led by parents and healthcare professionals, though some children made procedural choices, particularly regarding sedation. While allowing children to express preferences aligns with CCC’s emphasis on shared decision-making, their involvement was mostly limited to specific procedural aspects rather than broader treatment decisions.

Furthermore, decision-making should go beyond procedural choices to include meaningful involvement in aspects of care that are relevant to the child. CCC highlights that children should be empowered to make choices based on their needs and interests, not just within predefined boundaries. Expanding opportunities for children to engage in discussions about their treatment, such as preparation strategies, coping mechanisms, and pain management preferences, could enhance their role in decision-making while ensuring developmental appropriateness.

*Communication* played a key role in shaping children’s experiences of BoNT-A treatment. When explanations were clear and developmentally appropriate, children felt more secure, and parents emphasized that good communication helped reduce distress. However, there were variations in how information was conveyed, with healthcare providers being the primary source of information for both parents and children. While parents were engaged in supporting their child, there was limited evidence that they consistently mediated the information acquired from healthcare providers or actively facilitated discussions about the procedure.

In addition, communication should go beyond information delivery to actively foster dialog, expression, and co-creation. CCC highlights the importance of ensuring that children’s voices are heard and that they have opportunities to present their experiences and wishes. Moving towards a more interactive communication style, where children are encouraged to ask questions, express concerns, and participate in discussions, could strengthen their engagement. Training healthcare professionals in child-directed communication and encouraging parents to take an active role in discussions could further enhance children’s involvement in shaping their healthcare experiences.

By analyzing these findings through the CCC framework, this study highlights both strengths and areas for improvement in BoNT-A treatment for children with CP. While elements of CCC were evident, children’s participation and decision-making opportunities remained limited, and their perspectives were not systematically integrated into care decisions. Strengthening child-inclusive communication, structured preparation, and mechanisms for incorporating children’s experiences into treatment planning could enhance their involvement and better align care with CCC principles. Additionally, optimizing interaction within the triad of children, parents, and healthcare providers may facilitate a more cohesive approach to communication and decision-making, ensuring that children’s needs and perspectives are effectively acknowledged.

Table 5 provides an overview of example strategies that may support the implementation of CCC in the context of BoNT-A treatment. The strategies are organized according to the five CCC principles.

### 4.2. Discussion in Light of Earlier Research

The result from our study confirms that many children experience heightened anxiety in the days leading up to the BoNT-A treatment, and parents recognized the costs of the treatment for their child. This is in line with previous qualitative research, exploring parental perspectives on BoNT-A treatment for children with CP. Lorin and Forsberg [15] reported that many parents expressed anxiety and concerns about their child’s discomfort, while still complying with the treatment with the hope of improvements in muscle tone and mobility. This underscores the importance of promoting children’s agency and participation to ease the distress of the situation

Our results also reflect those of Karadag-Saygi et al. [14] who found that while many parents valued the positive effects of BoNT-A on their child’s function and comfort, they also weighed these benefits against the challenges of the procedure itself. In our study, parents described how clear communication and explanations from healthcare professionals helped reduce their child’s distress, mirroring previous findings that trust in medical staff and adequate information are essential for making the procedure feel more manageable. Furthermore, our study reinforces previous research showing that children develop individual coping strategies over time, with some becoming more resigned to the process, while others continue to experience distress with each new treatment [15,16].

Nguyen et al. [16] found that parents of nonambulatory children reported both positive and negative experiences with BoNT-A treatment, balancing its functional benefits against concerns about pain and distress during the procedure. Similarly, our findings highlight that while parents valued the potential benefits, they also observed heightened anxiety in their children before treatment and varying degrees of discomfort during the injections. However, while Nguyen et al. [16] primarily focused on parental reflections, our study provides additional insights by incorporating children’s own perspectives, offering a more comprehensive understanding of how BoNT-A treatment is experienced.

Taken together, these studies suggest that while BoNT-A is widely used and often seen as beneficial, the treatment process itself can be emotionally challenging for both children and their parents. This underscores the importance of child-centered communication, individualized support strategies, and preparation to optimize the overall experience of BoNT-A treatment.

### 4.3. Limitations

This study has some limitations that should be considered. First, the study design did not include longitudinal follow-up beyond four weeks post-treatment, limiting insights into how children’s perceptions and coping strategies evolve over multiple treatment cycles. Future research should explore long-term experiences with repeated BoNT-A interventions.

Second, as this study is a sub-study of an RCT, some children received placebo (saline) instead of BoNT-A [21]. While the injection procedure, dosage, and clinical setting were identical for both groups, experiences of treatment may still have been influenced by whether the child received active medication or placebo. However, given that procedural aspects such as the number and volume of the injections and preparation were the same, it is reasonable to assume that children’s immediate sensory and emotional experiences of the treatment were largely similar.

Third, the study was conducted within a specific healthcare setting, meaning the findings may reflect practices unique to this context. Differences in pain management, procedural explanations, and communication across institutions or countries could influence children’s and parents’ experiences. Comparative studies across different healthcare systems may provide further insights into these variations.

Fourth, this study only included children classified as GMFCS levels I and II. As such, the experiences of children with more severe cerebral palsy—who may undergo more invasive treatments and face different challenges—are not represented and warrant further research.

Fifth, a majority of the participants had previous experience with BoNT-A treatment. This may have influenced their perceptions, particularly in terms of familiarity with the procedure, access to relevant information, and the development of individualized pain management strategies. As such, prior experiences could have shaped both children’s and parents’ responses during the interviews, potentially limiting the generalizability of the findings to first-time treatment situations

Despite these limitations, the study offers valuable perspectives on how children and parents experience BoNT-A treatment, emphasizing the need for child-centered communication and support.

## 5. Conclusions

This study provides insight into how children with CP and their parents experience BoNT-A treatment, highlighting both the emotional and procedural challenges they face. The findings underscore the importance of clear, child-centered communication, structured pre-procedural preparation, and individualized support to minimize anxiety and enhance coping strategies. While some children adapted to the procedure over time, others continued to experience distress, emphasizing the need for approaches that foster participation, decision-making, and a sense of agency in a developmentally appropriate manner.

Additionally, the study highlights the triadic interaction among children, parents, and healthcare providers, where communication primarily occurred between healthcare professionals and each party individually, rather than facilitated as a shared triadic exchange. Optimizing this interaction by fostering more inclusive and child-sensitive communication strategies may help strengthen children’s engagement and ensure that their perspectives are more consistently integrated into care, in line with child-centered care principles.

To improve treatment experiences for children and their families, healthcare providers should integrate predictable routines, opportunities for children to express preferences, and a structured approach to engaging both parents and children in care discussions. Future research should explore long-term experiences with repeated BoNT-A treatments and evaluate interventions that actively incorporate children’s perspectives into treatment planning.

## Figures and Tables

**Table 1:** Interview guide.

Interview Questions
Knowledge about BoNT-A treatment
Can you describe what you know about Botox treatment?
What information did you receive beforehand?
How does the treatment work, and what is its purpose?
How is the procedure performed, including preparation and execution?
Experience of the treatment
Can you describe how you/your child experienced the treatment?
Post-treatment reflections
Can you describe how you/your child felt after the treatment?

**Table 2:** Principles of CCC.

Principle	Described as	Linked to
Agency	Described as being agentic, social agents, or agentic beings	Linked to children’s rights and their participation in healthcare
Participation	Described as degrees of involvement and inclusion	Linked to eliciting children’s perspectives and preferences
Impact	Described as impact on practice or experiences	Linked to context-specific and more generalizable impacts
Decision-making	Described in terms of children being involved	Linked to children making choices based on their needs and interests
Communication	Described in terms of voices, dialog, expression, and cocreation	Linked to presenting their experiences and wishes

**Table 3:** Demographics of participating children and parents.

Id	Age *	Gender	GMFCS	UL/BL	Parent	Earlier BoNT-A **
1	6	M	I	BL	mo	0
2	9	M	I	UL	fa	3
3	4	F	I	UL	mo	0
4	4	F	I	UL	fa	0
5	11	F	I	UL	mo	1
6	8	F	I	UL	mo	3
7	9	F	I	UL	fa	0
8	10	M	I	UL	mo	16
9	7	F	I	UL	fa	0
10	11	M	I	UL	fa	3
11	15	M	II	UL	fa	0
12	8	M	I	UL	mo	15
13	12	F	II	UL	mo	1
14	10	M	I	UL	mo	6
15	7	F	I	UL	mo	5
16	14	M	I	UL	fa	1 ^+^
17	8	M	I	UL	mo	1 ^+^
18	10	M	I	UL	fa	1 ^+^
19	9	F	I	UL	mo	0
20	10	F	II	UL	mo	9

Gender; male (M)/female (F), Gross Motor Function Classification System (GMFCS), unilateral (UL)/bilateral (BL), parent; mother (mo)/father (fa), * years; ** number of earlier treatments with Botulinum toxin A (BoNT-A); ^+^ more than one treatment (exact information not available).

**Table 4:** Themes and categories.

Themes	Categories
Preparing for the treatment	Between fear and familiarity
	Navigating information
Being in the moment	Managing pain
	Experiencing the injection
Adapting after treatment	Regaining stability
	Coping through rewards

**Table 5:** Example strategies supporting CCC in BoNT-A treatment.

CCC Principle	Example Strategies
Agency	- Use age-appropriate educational tools (e.g., booklets, animations) - Involve children in setting personal goals for treatment
Participation	- Provide structured time before and after treatment for questions - Use visual aids or choice boards to support expression of preferences - Include children in follow-up conversations about their experience
Impact	- Offer preparatory visits and opportunities to meet key staff - Address emotional impact through systematic feedback routines - Adjust procedures based on children’s reported experiences
Decision-making	- Let children participate in planning appointment schedules - Identify preferred coping strategies (e.g., music, toys) - Review and adjust treatment plans based on prior experiences
Communication	- Use developmentally appropriate language - Encourage children to participate directly in discussions - Use role-play or visual storytelling to explain procedures - Support parents in continuing conversations at home

## Data Availability

The data presented in this study are available on request from the corresponding author. The data are not publicly available due to ethical reasons; public sharing of data was not specifically consented for by participants.
